# Autocrine TGF-β1 Maintains the Stability of Foxp3^+^ Regulatory T Cells via IL-12Rβ2 Downregulation

**DOI:** 10.3390/biom10060819

**Published:** 2020-05-27

**Authors:** Garam Choi, Hyeongjin Na, Da-Sol Kuen, Byung-Seok Kim, Yeonseok Chung

**Affiliations:** 1 Laboratory of Immune Regulation, Research Institute of Pharmaceutical Sciences, College of Pharmacy, Seoul National University, Seoul 08826, Korea; grhappy@snu.ac.kr (G.C.); hyeongjinna@snu.ac.kr (H.N.); dkuen592@snu.ac.kr (D.-S.K.); 2 BK21 Plus Program, College of Pharmacy, Seoul National University, Seoul 08826, Korea; 3 Division of Life Sciences, College of Life Science and Bioengineering, Incheon National University, Incheon 22012, Korea

**Keywords:** regulatory T cell, stability, TGF-β1, IL-12R

## Abstract

Transforming growth factor beta 1 (TGF-β1) is an immunosuppresive cytokine that plays an essential role in immune homeostasis. It is well known that regulatory T (Treg) cells express TGF-β1; however, the role of autocrine TGF-β1 in the development, function, and stability of Treg cells remains poorly understood. We found that Treg cell-derived TGF-β1 was not required for the development of thymic Treg cells in mice, but played a role in the expression of latency-associated peptide and optimal suppression of naïve T cell proliferation in vitro. Moreover, the frequency of Treg cells was significantly reduced in the mesenteric lymph nodes of the Treg cell-specific TGF-β1-deficient mice, which was associated with increased frequency of IFN-γ-producers among Treg cells. TGF-β1-deficient Treg cells were more prone to express IFN-γ than TGF-β1-sufficient Treg cells in a dendritic cell-mediated stimulation in vitro as well as in an adoptive transfer study in vivo. Mechanistically, TGF-β1-deficient Treg cells expressed higher levels of *Il12rb2* and were more sensitive to IL-12-induced conversion into IFN-γ-producing Treg cells or IFN-γ-producing exTreg cells than TGF-β1-sufficient Treg cells. Our findings demonstrate that autocrine TGF-β1 plays a critical role in the optimal suppressive activity and stability of Treg cells by downregulating IL-12R on Treg cells.

## 1. Introduction

Regulatory T (Treg) cells are a heterogeneous subset of helper T cells that express the forkhead box transcription factor Foxp3 and play an essential role in the maintenance of peripheral tolerance and suppression of excessive immune responses [1,2,3,4,5]. Treg cells constitute 10–15% of CD4^+^ T cells in the secondary lymphoid organs and can be categorized into thymus-derived Treg (tTreg) cells and peripheral Treg (pTreg) cells based on their developmental origin [6,7,8]. Helios and Neuropilin-1 (Nrp1) have been suggested as specific markers for thymic Treg cells [9,10].

TGF-β is a pleiotropic cytokine that regulates a number of biological processes including embryogenesis, wound healing, angiogenesis, and immune regulation. In mammals, TGF-β has three different isoforms, TGF-β1, TGF-β2, and TGF-β3, of which TGF-β1 serves as the predominant form found in immune cells [11]. TGF-β1-deficient mice develop excessive multi-organ autoimmune phenotypes at an early age and die within 3–4 weeks after birth [12,13,14], indicating an essential role of TGF-β1 in preventing autoimmunity in the periphery. TGF-β1 is synthesized in an inactive precursor form composed of a dimer of mature TGF-β1 that is surrounded by latency-associated peptide (LAP) [15]. Activation of latent TGF-β1 by the engagement of integrin, such as αvβ8, expressed on myeloid cells [16,17], releases mature TGF-β1 after proteolysis of LAP [18,19].

TGF-β1 signaling is known to be tightly associated with the development, function and stability of Treg cells. Although earlier studies suggested that TGF-β1 is dispensable for the development of tTreg cells [20,21], a recent study demonstrated that TGF-β1 supports early Treg cell development in the thymus by antagonizing negative selection [22]. Moreover, TGF-β1 is essential for the differentiation of Treg cells from naïve CD4 T cells in the periphery. TGF-β1 production by Treg cells and TGF-β signaling in Treg cells have been shown to be required for Treg cell-mediated suppression in vivo. For example, by using Foxp3^+^ cell-specific TGF-β1-deficient mice, it was shown that TGF-β1 from Treg cells is required for the suppression of type 1 helper T (Th1)-dependent intestinal inflammation [23,24], whereas TGF-βRII signaling in Treg cells is required for Treg cell suppression of Th17 immune responses in the colon [25].

Accumulating evidence shows that Treg cells can gain effector function and contribute to the pathogenesis of inflammatory diseases by producing effector cytokines of Th1 cells (IFN-γ) or Th17 cells (IL-17) [17,26,27,28,29] or by transdifferentiating into follicular helper T (Tfh) cells in the Peyer’s patches [30]. For instance, *Toxoplasma gondii* infection has been shown to increase the expression of T-bet and IFN-γ in Treg cells in mice [31], and patients with type 1 diabetes (T1D) have a higher number of IFN-γ-producing Treg cells than healthy individuals [32]. On the other hand, IL-6 was shown to stimulate Treg cells to produce IL-17-producing Treg cells which exhibit significantly diminished suppressive activity [33,34,35]. Hence, the stability and plasticity of Treg cells are significantly impacted by the cytokines produced in the inflammation site [36,37]. Several findings indicate that differentiation of naïve Treg cells is driven by transcription factors including T-bet, IRF4, RORγt, STAT3, and Bcl6, which are essential for the differentiation of conventional CD4^+^ T cells [38,39,40,41,42,43,44]. However, detailed molecular mechanisms that control the functional specialization and differentiation of effector cytokine-producing Treg cells are poorly understood.

In this study, we address the roles of Treg cell-derived TGF-β1 in the development and stability of Treg cells by using multiple in vivo models. We demonstrate that autocrine TGF-β1 plays little role in the development of thymic Treg cells and that TGF-β1-deficient Treg cells exhibit a slightly diminished suppressive activity in vitro. Notably, TGF-β1-deficient Treg cells harbor increased frequency of IFN-γ^+^ cells in the mesenteric lymph nodes (MLN) in steady state. Mechanistic studies showed that TGF-β1-deficient Treg cells are less resistant to become IFN-γ-producers upon IL-12, but not IL-27, stimulation, and that autocrine TGF-β1 is required for suppression of *Il12rb2* expression in Treg cells. Collectively, our findings provide a crucial role for autocrine TGF-β1 in maintaining the stability and function of Treg cells.

## 2. Materials and Methods

### 2.1. Ethics Statement

All animal experiments were approved by the Institutional Animal Care and Use Committee of Seoul National University (IACUC protocol number: SNU-160422-3) and were performed in accordance with guidelines of Seoul National University for the care and use of laboratory animals.

### 2.2. Mice

B6.SJL and *Rag1^−/−^* mice were purchased from Jackson Laboratory (Bar Harbor, ME, USA). *Tgfb1^fl/fl^* mice and *Cd4Cre* mice were kindly provided by Drs. Ming O. Li (Memorial Sloan Kettering Cancer Center, New York, NY, USA) and Chen Dong (Tsinghua University, Beijing, China), respectively. *Foxp3^YFP-Cre^* mice were kindly provided by Dr. Jae-Hoon Chang (Yeungnam University, Gyeongsan, Korea). *Tgfb1^fl/fl^* mice were crossed with *Foxp3^YFP-Cre^* or *Cd4Cre* mice for in vivo and in vitro studies. All mice were maintained in the Animal Center for Pharmaceutical Research of Seoul National University under specific-pathogen free conditions.

### 2.3. Flow Cytometric Analysis

For CD4 T cell analysis, thymus, spleen, peripheral lymph nodes, and mesenteric lymph nodes were isolated from 8- to 12-week-old *Foxp3^YFP-Cre^Tgfb1^fl/+^*, *Foxp3^YFP-Cre^Tgfb1^fl/fl^*, *Tgfb1^fl/fl^*, and *Cd4CreTgfb1^fl/fl^* mice. The cells from the mice were stained with BUV737-conjugated anti-mouse CD4 (BD Biosciences, San Jose, CA, USA), BV510-conjugated anti-mouse CD4 (Biolegend, San Diego, CA, USA), APC/Cy7-conjugated anti-mouse CD45.1 (Biolegend, San Diego, CA, USA), PerCP/Cy5.5-conjugated anti-mouse CD45.1 (Biolegend, San Diego, CA, USA), BUV395-conjugated anti-mouse-CD45.2 (BD Biosciences, San Jose, CA, USA), APC/Cy7-conjugated anti-mouse CD45.2 (Biolegend, San Diego, CA, USA), eFlour450-conjugated anti-mouse Foxp3 (Thermo Fisher Scientific, Waltham, MA, USA), Alexa Flour^TM^ 647-conjugated anti-mouse Foxp3 (Biolegend, San Diego, CA, USA), PerCP/Cy5.5-conjugated anti-mouse CD304 (Nrp1) (Biolegend, San Diego, CA, USA), PE/Cy7-conjugated anti-mouse/rat CD278 (ICOS) (Biolegend, San Diego, CA, USA), FITC-conjugated anti-mouse CD279 (PD-1) (Thermo Fisher Scientific, Waltham, MA), PE-conjugated anti-mouse CD357 (GITR) (Biolegend, San Diego, CA, USA), and APC-conjugated anti-mouse CD152 (CTLA4) (Thermo Fisher Scientific, Waltham, MA, USA). For intracellular staining, the cells were incubated for 3–6 h with 100 ng/mL of PMA (Sigma Aldrich, Saint Louis, MO, USA), 1 µM of ionomycin (Sigma Aldrich, Saint Louis, MO, USA), brefeldin A, and monensin (Thermo Fisher Scientific, Waltham, MA, USA). After incubation, the cells were fixed and permeabilized using a Foxp3/Transcription Factor Staining Buffer Set (Thermo Fisher Scientific, Waltham, MA, USA) and were stained with PE-conjugated anti-mouse LAP (TGF-β1) (Biolegend, San Diego, CA, USA), APC-conjugated anti-mouse LAP (TGF-β1) (Biolegend, San Diego, CA, USA), PE/Cy7-conjugated anti-mouse TIGIT (Vstm3) (Biolegend, San Diego, CA, USA), PE-conjugated anti-mouse IFN-γ (Biolegend, San Diego, CA, USA), PE/Cy7-conjugated anti-mouse IFN-γ (Biolegend, San Diego, CA, USA), BUV395-conjugated anti-mouse IL-17A (BD Biosciences, San Jose, CA, USA), Alexa Fluor 647-conjugated anti-mouse IL-4 (Biolegend, San Diego, CA, USA), and PE-conjugated anti-mouse IL-17A (Biolegend, San Diego, CA, USA). These cells were analyzed by LSRFortessa^TM^ or FACSLyric^TM^ (BD Biosciences, San Jose, CA, USA), and obtained data were analyzed using Flowjo software (BD Biosciences, San Jose, CA, USA).

### 2.4. In Vitro Differentiation

CD4 T cells were isolated from lymph nodes and spleens of *Foxp3^YFP-Cre^Tgfb1^fl/+^* or *Foxp3^YFP-Cre^Tgfb1^fl/fl^* mice with a CD4^+^ T Cell Isolation Kit (Miltenyi Biotec, Bergisch Gladbach, Germany), and then naïve CD4 T cells (CD4^+^CD25^−^CD44^low^CD62L^high^) were sorted using FACSAria^TM^ III (BD Biosciences, San Jose, CA, USA). For Treg cell differentiation, anti-CD3ε (2C11, 1 µg/mL) (BioXcell, West Lebanon, NH, USA) and anti-CD28 (37.51, 1 µg/mL) (BioXcell, West Lebanon, NH, USA) Abs were pre-coated in a 96-well flat bottom plate (Corning, Steuben Country, NY, USA) overnight at 4 °C. After washing the plate with phosphate-buffered saline (GenDEPOT, Katy, TX, USA), naïve CD4 T cells (1 × 10^5^ cells/well) were cultured with IL-2 (20 ng/mL) (PeproTech, Rocky Hill, NJ, USA), TGF-β1 (3 ng/mL) (PeproTech, Rocky Hill, NJ, USA), and anti-IFN-γ (XMG1.2, 1 µg/mL) (BioXcell, West Lebanon, NH, USA) for 96 h.

### 2.5. In Vitro Suppression Assay

For Treg cell suppression assay, CD4^+^YFP^+^ Treg cells were isolated from the spleens of *Foxp3^YFP-Cre^Tgfb1^fl/+^* (CD45.2^+/+^) or *Foxp3^YFP-Cre^Tgfb1^fl/fl^* mice (CD45.2^+/+^) using the CD4^+^ T Cell Isolation Kit and FACSAria^TM^ III. Naïve CD4 T cells isolated from B6.SJL (CD45.1^+/+^) mice were labeled with 2 µM of the CellTrace^TM^ Violet (CTV) Cell Proliferation Kit (Thermo Fisher Scientific, Waltham, MA, USA) for 15 min at 37 °C. CTV-labeled naïve T cells (5 × 10^4^ cells/well) were co-cultured with CD4^+^YFP^+^ Treg cells at an indicated ratio in the presence of anti-CD3ε (1 µg/mL) and irradiated splenocytes. After 96 h, the cells were harvested and stained with fluorescent dye-conjugated antibodies. Percentage suppression was calculated using the following formula:

100−((Divided cell % of CD45.1^+/+^ cells treated with Treg/Divided cell % of CD45.1^+/+^ alone) × 100)

### 2.6. In Vivo Adoptive T Cell Transfer

CD4^+^YFP^+^ Treg cells (1 × 10^6^ cells/injection) isolated from *Foxp3^YFP-Cre^Tgfb1^fl/+^* or *Foxp3^YFP-Cre^Tgfb1^fl/fl^* mice and naïve CD4 T cells (1 × 10^6^ cells/injection) isolated from B6.SJL mice were intravenously transferred into *Rag1^−/−^* mice. Four weeks after the transfer, all mice were euthanized and the spleens and mesenteric lymph nodes were collected for further analysis.

### 2.7. In Vitro Treg Cell Conversion Assay

CD4^+^YFP^+^ Treg cells (1 × 10^5^ cells/well) isolated from *Foxp3^YFP-Cre^Tgfb1^fl/+^* or *Foxp3^YFP-Cre^Tgfb1^fl/fl^* mice were co-cultured with bone marrow-derived dendritic cells (BMDC, 2 × 10^4^ cells/well) in the presence of LPS (100 ng/mL) (Sigma Aldrich, Saint Louis, MO, USA) for 72 h. For BMDC-free Treg cell conversion, CD4^+^YFP^+^ Treg cells (1 × 10^5^ cells/well) were stimulated with IL-6 (PeproTech, Rocky Hill, NJ, USA), IL-12 (PeproTech, Rocky Hill, NJ, USA) or IL-27 (R&D Systems, Minneapolis, MN, USA) in 96-well plates pre-coated with anti-CD3ε (1 µg/mL). After 72 h, the cells were treated with PMA, ionomycin, brefeldin A, and monensin for 3–6 h. The cells were harvested and stained for flow cytometric analysis. To examine the role of TGF-β1 in Treg cells, various concentrations of TGF-β1 (0.2, 1, 5, and 25 ng/mL) were treated to CD4^+^YFP^+^ Treg cells (1 × 10^5^ cells/well).

### 2.8. Quantitative RT-PCR

To investigate gene expression in Treg or Foxp3^−^CD4^+^ T cells, we isolated total RNA from FACS-sorted CD4^+^YFP^+^ (Treg) or CD4^+^YFP^−^ (Foxp3^−^CD4^+^ T) cells using TRIzol Reagent (Thermo Fisher Scientific, Waltham, MA, USA) and synthesized cDNA from the RNA using RevertAid First Strand cDNA Synthesis Kit (Thermo Fisher Scientific, Waltham, MA, USA) according to the protocol provided by the manufacturer. Quantitative RT-PCR (qRT-PCR) was performed using SYBR Green Supermix (Bio-Rad, Hercules, CA, USA) and Applied Biosystems 7500 Fast real-time PCR System (Applied Biosystems, Foster City, CA, USA). Primers for mouse *Actb* (Forward: 5′-TGG AAT CCT GTG GCA TCC ATG AAA C-3′, Reverse: 5′-TAA AAC GCA GCT CAG TAA CAG TCC G-3′), *Tgfb1* (Forward: 5′-GCA ACA TGT GGA ACT CTA CCA GA-3′, Reverse: 5′-GAC GTC AAA AGA CAG CCA CTC A-3′), *Tbx21* (Forward: 5′-CAA CAA CCC CTT TGC CAA AG-3′, Reverse: 5′-TCC CCC AAG CAG TTG ACA GT-3′), *Ifng* (Forward: 5′-GAT GCA TTC ATG AGT ATT GCC AAG T-3′, Reverse: 5′-GTG GAC CAC TCG GAT GAG CTC-3′), *Il12rb1* (Forward: 5′-CCC CAG CGC TTT AGC TTT-3′, Reverse: 5′-GCC AAT GTA TCC GAG ACT GC-3′), *Il12rb2* (Forward: 5′-TGT GGG GTG GAG ATC TCA GT-3′, Reverse: 5′-TCT CCT TCC TGG ACA CAT GA-3′), *Ifngr1* (Forward: 5′-TCA AAA GAG TTC CTT ATG TGC CT-3′, Reverse: 5′-TAC GAG GAC GGA GAG CTG TT-3′), *Ifngr2* (Forward: 5′-TCC TGT CAC GAA ACA ACA GC-3′, Reverse: 5′-ACA TCC AAT GTT GCT GCT GT-3′), *Il27ra* (Forward: 5′-CAA GAA GAG GTC CCG TGC TG-3′, Reverse: 5′-TTG AGC CCA GTC CAC CAC AT -3′) and *Il6st* (Forward: 5′- ATA GTC GTG CCT GTG TGC TTA-3′, Reverse: 5′-GGT GAC CAC TGG GCA ATA TG-3′) were purchased from Macrogen (Seoul, Korea) and Cosmogenetech (Seoul, Korea). Relative gene expression was normalized to β-actin (*Actb*).

### 2.9. Statistical Analysis

Data were analyzed with GraphPad Prism v8 (GraphPad Software, San Diego, CA, USA). *P* values were determined using two-tailed Student’s *t*-test and are presented within each figure and figure legend.

## 3. Results

### 3.1. The Role of Autocrine TGF-β1 in the Development of Thymic and Peripheral Treg Cells

**Figure 1:**
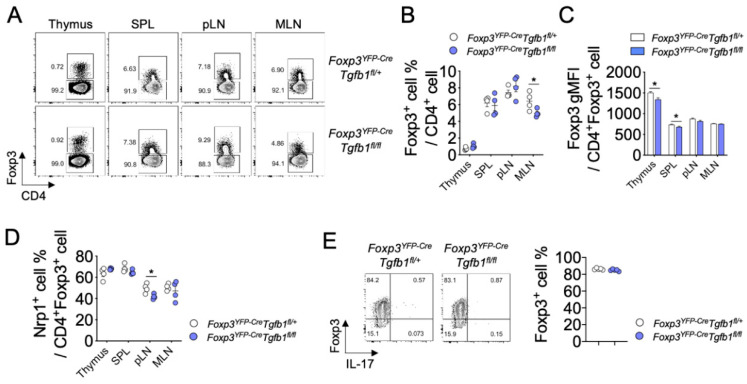
Autocrine TGF-β1 is dispensable for the development and differentiation of Treg cells. (**A**) Flow cytometric analysis of Foxp3 expression in CD4^+^ T cells from the thymus, spleen (SPL), peripheral lymph nodes (pLN), and mesenteric lymph nodes (MLN) of *Foxp3^YFP-Cre^Tgfb1^fl/+^* and *Foxp3^YFP-Cre^Tgfb1^fl/fl^* mice at 8 weeks (*n* = 4). (**B**,**C**) Graph depicting the frequency and geometric mean fluorescence intensity (gMFI) of Foxp3^+^ cells among gated CD4^+^ cells from thymus, SPL, pLN and MLN (*n* = 4). (**D**) Expression of Nrp1 in CD4^+^Foxp3^+^ cells from thymus, SPL, pLN, and MLN (*n* = 4). (**E**) Naïve T cells from *Foxp3^YFP-Cre^Tgfb1^fl/fl^* and control mice were differentiated to Treg cells in vitro for three days and analyzed for the expression of Foxp3 by flow cytometry. Data are representative of three (**E**) or four (**A**–**D**) independent experiments. Quantification plots show mean ± SD (**B**,**D**,**E**) and mean + SEM (**C**). * *p* ≤ 0.05 based on two-tailed Student’s *t*-test.

To investigate the role of Treg cell-derived TGF-β1 in the development, function, and plasticity of Treg cells, we generated Treg cell-specific *Tgfb1* conditional knockout mice by crossing *Foxp3^YFP-Cre^* knock-in mice that express the yellow fluorescent protein (YFP)/iCre-recombinase fusion protein under the control of *Foxp3* locus with *Tgfb1^fl/fl^* mice (*Foxp3^YFP-Cre^Tgfb1^fl/fl^*). As expected, the level of *Tgfb1* was significantly diminished in Foxp3^+^CD4^+^ Treg cells from *Foxp3^YFP-Cre^Tgfb1^fl/fl^* mice compared to that from *Foxp3^YFP-Cre^Tgfb1^fl/+^* mice, while it remained comparable in Foxp3^−^CD4^+^ T cells between the two groups, indicating a Treg cell-specific deletion of the *Tgfb1* gene in *Foxp3^YFP-Cre^Tgfb1^fl/fl^* mice (Figure S1A). In parallel with this observation, we found that the cell surface expression of LAP (TGF-β1), a latent form of TGF-β1, was significantly diminished in Foxp3^+^CD4^+^ Treg cells, but not in Foxp3^−^CD4^+^ T cells, from *Foxp3^YFP-Cre^Tgfb1^fl/fl^* mice compared to that from *Foxp3^YFP-Cre^Tgfb1^fl/+^* littermate controls (Figure S1B). The *Foxp3^YFP-Cre^Tgfb1^fl/fl^* mice did not show any sign of spontaneous development of inflammatory diseases up to 12 months after birth. We observed comparable frequencies of Foxp3^+^ cells among CD4^+^ T cells in the thymus as well as in the spleen and peripheral lymph nodes (pLN) between *Foxp3^YFP-Cre^Tgfb1^fl/fl^* and littermate control mice. However, we observed a moderate but significant decrease in the frequency of Foxp3^+^ Treg cells in the MLN of *Foxp3^YFP-Cre^Tgfb1^fl/fl^* mice (Figure 1A,B). On the other hand, the intensity of Foxp3 expression in Treg cells was slightly lower in the thymus and spleen, but not in the lymph nodes, in *Foxp3^YFP-Cre^Tgfb1^fl/fl^* mice (Figure 1C). *Cd4CreTgfb1^fl/fl^* mice, a T cell-specific *Tgfb1* conditional knockout mouse strain, exhibited an increased frequency of thymic Treg cells without any change in Treg cell frequencies in the periphery (Figure S2A,B).

Cell surface expressions of Nrp1 and Helios on Treg cells have been suggested to be associated with tTreg/pTreg distinction or Treg cell stability [10]. To determine whether TGF-β1-deficiency in Treg cells affects the frequency of Nrp1^+^ or Helios^+^ Treg cells, we analyzed Nrp1 and Helios expressions on Treg cells. The frequencies of Nrp1^+^ Treg cells in the thymus, spleen, and MLN were comparable between the two groups, except for a significant decrease in the pLNs from *Foxp3^YFP-Cre^Tgfb1^fl/fl^* mice (Figure 1D). A similar decrease in Nrp1 expression on TGF-β1-deficient Treg cells in *Cd4CreTgfb1^fl/fl^* mice was evident in the MLN (Figure S2C). In contrast to the frequencies of Nrp1, frequencies of Helios^+^ Treg cells were uniformly increased in all three secondary lymphoid organs of *Foxp3^YFP-Cre^Tgfb1^fl/fl^* mice (Figure S3A).

Naïve CD4^+^ T cells can be differentiated into Foxp3^+^ pTreg cells or in vitro-induced Treg (iTreg) cells upon TGF-β1 stimulation in vitro and in vivo. We stimulated naïve CD4^+^ T cells isolated from *Foxp3^YFP-Cre^Tgfb1^fl/fl^* or *Foxp3^YFP-Cre^Tgfb1^fl/+^* mice in an in vitro Treg cell differentiation system with exogenous TGF-β1 and found a comparable frequency of Foxp3^+^ Treg cells between the two groups (Figure 1E). Similarly, a comparable iTreg cell induction was observed between naïve CD4^+^ T cells of *Cd4CreTgfb1^fl/fl^* and *Tgfb1^fl/fl^* mice (Figure S2D). These data suggest that Treg cell-derived TGF-β1 is dispensable for the thymic development or the peripheral differentiation of Treg cells but is required for homeostasis of Treg cells in the microenvironment of MLN.

### 3.2. The Role of Autocrine TGF-β1 in the Phenotype and Suppressive Function of Treg Cells

**Figure 2:**
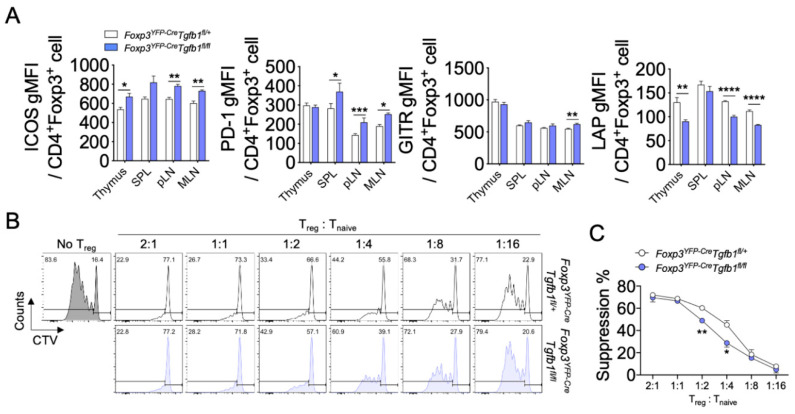
Suppressive capacity and cytokine production of TGF-β1-deficient Treg cells. (**A**) Expression of ICOS, PD-1, GITR, and LAP in CD4^+^Foxp3^+^ cells from the thymus, SPL, pLN, and MLN (*n* = 4). (**B**) Treg cell suppression assay. Sorted Treg cells (CD45.2^+^) from *Foxp3^YFP-Cre^Tgfb1^fl/+^* and *Foxp3^YFP-Cre^Tgfb1^fl/fl^* mice were cultured with CellTrace Violet (CTV)-labeled naïve T cells (CD45.1^+^) and irradiated splenocytes in the presence of anti-CD3ε. The percentages of divided T cells are shown in each histogram plot. (**C**) Graph depicting the percentage of suppression among gated CD45.1^+^ cells. Data are representative of three (**B**,**C**) or four (**A**,**D**) independent experiments. * *p* ≤ 0.05, ** *p* ≤ 0.01, *** *p* ≤ 0.001, and **** *p* ≤ 0.0001 based on two-tailed Student’s *t*-test.

Autocrine TGF-β1 has been suggested to be required for Treg cells to efficiently suppress the proliferation, activation, and differentiation of effector T cells [21,23]. To determine whether the lack of autocrine TGF-β1 impacts the phenotype and function of Treg cells, we compared the levels of TIGIT, CTLA4, ICOS, PD-1, and GITR, all of which are associated with suppressive capacity or activation status of Treg cells between Treg cells from *Foxp3^YFP-Cre^Tgfb1^fl/fl^* and *Foxp3^YFP-Cre^Tgfb1^fl/+^* mice. We observed no evident differences in the levels of TIGIT and CTLA4 between TGF-β1-sufficient and TGF-β1-deficient Treg cells (Figure S3B). The levels of ICOS and PD-1 were higher in the TGF-β1-deficient Treg cells than in the TGF-β1-sufficient Treg cells (Figure 2A). The expression of GITR on Treg cells was comparable between the two groups, except for a slight increase in the TGF-β1-deficient Treg cells in the MLN (Figure 2A). By contrast, the surface expression levels of LAP on Treg cells were all significantly decreased in the thymus, pLN, and MLN of the former group (Figure 2A).

To directly determine the role of autocrine TGF-β1 in the suppressive function of Treg cells, we performed an in vitro suppression assay by co-culturing CellTrace Violet dye-labeled naïve CD4^+^ T cells with FACS-sorted YFP^+^ Treg cells from *Foxp3^YFP-Cre^Tgfb1^fl/fl^* or *Foxp3^YFP-Cre^Tgfb1^fl/+^* littermate control mice in the presence of anti-CD3ε and irradiated splenocytes. A different ratio of Treg cell to naïve CD4^+^ T cell (T_naive_) was added to the culture to determine whether Treg cells from TGF-β1-sufficient and TGF-β1-deficient mice had different suppressive capacities. As shown in Figure 2B,C, suppression was significantly reduced in TGF-β1-deficient Tregs when a ratio of 1:2 and 1:4 was applied to the culture. Collectively, Treg cells lacking TGF-β1 had heightened activated phenotypes, lower surface LAP expression, and less suppressive activity on effector T cells.

### 3.3. Autocrine TGF-β1 Contributes to the Stability of Treg Cells

**Figure 3:**
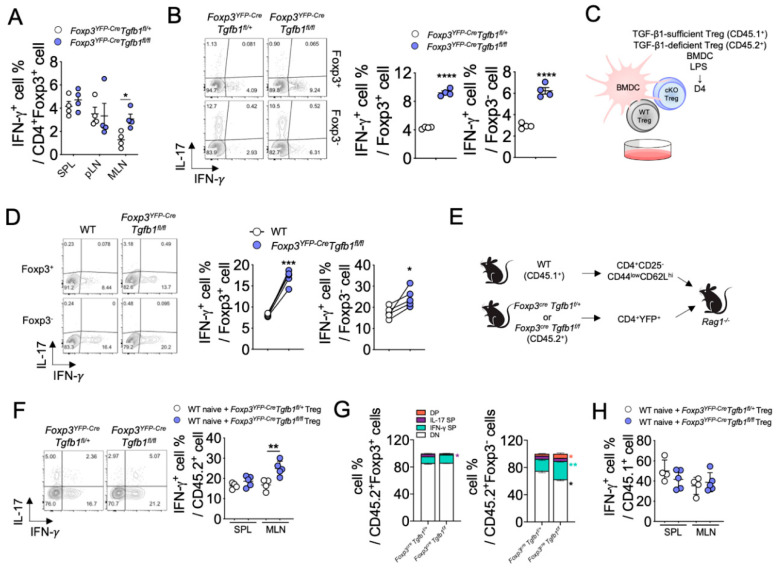
TGF-β1 deficiency intrinsically promotes the conversion of Treg cells into IFN-γ-producing cells. (**A**) Quantification of IFN-γ-expressing cells within CD4^+^ cells from SPL, pLN, and MLN (*n* = 4). (**B**) Purified Treg cells co-cultured with bone marrow-derived dendritic cells (BMDC) in the presence of LPS. Representative contour plots of IFN-γ- and IL-17A-expressing cells within CD4^+^Foxp3^+^ or CD4^+^Foxp3^−^ cells. Quantification of IFN-γ-expressing cells among CD4^+^Foxp3^+^ or CD4^+^Foxp3^−^ cells. (**C**,**D**) Mixed-Treg cell co-culture experiment. (**C**) Schematic representation of mixed-Treg cell co-culture experiment. (**D**) Flow cytometric analysis of IFN-γ and IL-17 expression in CD45.1^+^ (wild-type (WT); TGF-β1-sufficient) or CD45.2^+^ (conditional knockout (cKO); TGF-β1-deficient) cells. (**E**) Schematic representation of Treg cell adoptive co-transfer experiment. WT naïve T cells (CD45.1^+^) were transferred to *Rag1^−/−^* mice with sorted YFP^+^Tregs cells from *Foxp3^YFP-Cre^Tgfb1^fl/+^* or *Foxp3^YFP-Cre^Tgfb1^fl/fl^* mice (*n* = 4–5). (**F**) Flow cytometry plots of IFN-γ and IL-17 expression in MLN of CD45.2^+^ cells and quantification of IFN-γ^+^ cells among CD45.2^+^ cells in SPL and MLN (*n* = 4–5). (**G**) Proportion of IFN-γ- or IL-17-producing cells among Foxp3^−^CD45.2^+^ or Foxp3^+^CD45.2^+^ cells in MLN (*n* = 4–5). (**H**) Quantification of IFN-γ^+^ cells among CD45.1^+^ cells in SPL and MLN (*n* = 4–5). Data are representative of four (**A**), five (**B**), or two (**C**–**H**) independent experiments. Quantification plots show mean ± SD (**A**,**B**,**F**,**H**). * *p* ≤ 0.05, ** *p* ≤ 0.01, *** *p* ≤ 0.001, and **** *p* ≤ 0.0001 based on two-tailed Student’s *t*-test.

Treg cells are known to differentiate into IFN-γ- or IL-17-producing Treg cells, or to transdifferentiate into Foxp3-negative Th1, Th17 or Tfh cells, referred to as exTreg cells, under lymphopenic or inflammatory conditions in mice [26,29], and in patients with cancer, autoimmune disease or infections [17,27,28,45,46,47]. To determine if autocrine TGF-β1 has a role in maintaining the stability of Treg cells, we examined the frequency of IFN-γ-, IL-4-, or IL-17-producers among Foxp3^+^ Treg cells in *Foxp3^YFP-Cre^Tgfb1^fl/fl^* mice. Of interest, a lack of autocrine TGF-β1 in *Foxp3^YFP-Cre^Tgfb1^fl/fl^* mice showed an increased frequency of IFN-γ^+^ Treg cells in the MLNs, but not in other secondary lymphoid organs, compared to littermate control mice under steady state while the frequencies of IL-4^+^ and IL-17^+^ Treg cells remained comparable between the two groups (Figure 3A and Figure S4A). These observations suggest that Treg cell-derived TGF-β1 promotes the stability of Treg cells by inhibiting the IFN-γ production from Treg cells in the MLN.

To further investigate the impact of Treg cell-derived TGF-β1 on Treg cell stability, we isolated Treg cells from *Foxp3^YFP-Cre^Tgfb1^fl/fl^* or littermate control mice and co-cultured them with bone marrow-derived dendritic cells (BMDCs) in the presence of anti-CD3ε and LPS for three days. We observed a significantly higher frequency of IFN-γ^+^ cells among Foxp3^+^ cells in the TGF-β1-deficient Treg cells compared to TGF-β1-sufficient Treg cells (Figure 3B). Similarly, the frequency of IFN-γ^+^ cells among Foxp3^−^ exTreg cells was also significantly higher in the former group (Figure 3B). However, the frequency of IL-17^+^ cells among Foxp3^+^ cells or Foxp3^−^ exTreg cells was comparable between TGF-β1-deficient and TGF-β1-sufficient Treg cells (Figure S4B). To determine whether the observed increase in IFN-γ production by TGF-β1-deficient Treg cells was due to deficiency of autocrine TGF-β1, we performed a similar experiment with mixed TGF-β1-sufficient Treg (CD45.1^+^) and TGF-β1-deficient Treg cells (CD45.2^+^) (Figure 3C). We observed significantly higher frequencies of both IFN-γ^+^ and IL-17^+^ cells in TGF-β1-deficient Treg cells than in TGF-β1-sufficient Treg cells, among Foxp3^+^ populations as well as in Foxp3^−^ exTreg populations (Figure 3D and Figure S4C).

To further explore the role of autocrine TGF-β1 in Treg cell stability in vivo, we co-transferred naïve CD4^+^ T cells (CD45.1^+^) with either TGF-β1-sufficient or TGF-β1-deficient Treg cells (CD45.2^+^) into *Rag1^−/−^* mice (Figure 3E). Consistent with the in vitro experiments in Figure 3B, we observed that a significantly higher frequency of IFN-γ^+^ cells was induced from TGF-β1-deficient Treg cells compared to TGF-β1-sufficient Treg cells (Figure 3F), but there was no significant difference in the frequency of IL-17^+^ cells between the two groups (Figure S4D). To determine whether Treg cell-specific TGF-β1 deficiency induces the conversion of Treg cells into exTreg or Th1-like Treg cells, we analyzed the percentage of IFN-γ^+^ cells among Foxp3^−^ or Foxp3^+^ donor cells. As shown in Figure 3G, only Foxp3^−^ exTreg cells from TGF-β1-deficient cells expressed a higher level of IFN-γ than TGF-β1-sufficient donor cells. The frequencies of IFN-γ^+^ cells derived from CD45.1^+^ naïve CD4^+^ T cells were comparable between the two groups (Figure 3H). Collectively, these findings demonstrate that autocrine TGF-β1 contributes to the stability of Treg cells by restraining conversion into IFN-γ-producing T cells.

### 3.4. Autocrine TGF-β1 Maintains the Stability of Treg Cells by Inhibiting IL-12Rβ Expression

**Figure 4:**
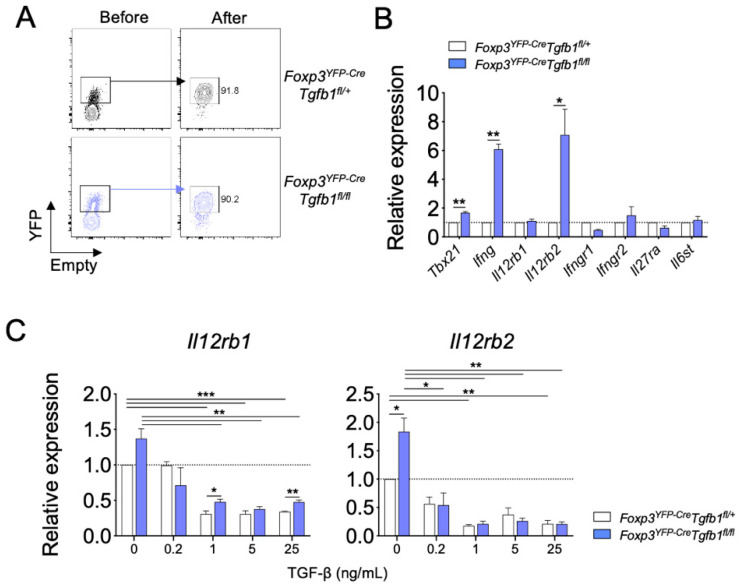
TGF-β1 regulates IFN-γ production of Treg cells by downregulating IL-12 signaling. (**A**) Sorting strategy and purity of Treg cells from *Foxp3^YFP-Cre^Tgfb1^fl/+^* and *Foxp3^YFP-Cre^Tgfb1^fl/fl^* mice. (**B**) Sorted Treg cells were stimulated anti-CD3ε and analyzed for Th1 response-related gene expression by qRT-PCR (*n* = 3). (**C**) Sorted Treg cells were cultured with TGF-β1 (0.2, 1, 5, and 25 ng/mL) in the presence of anti-CD3ε and IL-12, and the relative *Il12rb1* and *Il12rb2* expression was analyzed by qRT-PCR. Data are representative of three independent experiments, and values are expressed as the mean +SEM (**B**,**C**). * *p* ≤ 0.05, ** *p* ≤ 0.01, and *** *p* ≤ 0.0001 compared to control Treg cells based on Student’s *t*-test.

Proinflammatory cytokines, such as IFN-γ, IL-12, and IL-27, have been recognized as potent inducers of IFN-γ production in CD4^+^ T cells. To uncover molecular mechanisms by which autocrine TGF-β1 inhibits the conversion of Treg cells into IFN-γ^+^ cells, we compared the expression of cytokine receptors, including IL-12Rβ1, IL-12Rβ2, IFN-γR1, IFN-γR2, IL-27Rα, and gp130, between TGF-β1-sufficient and TGF-β1-deficient Treg cells (Figure 4A). In accordance with the observed elevation in IFN-γ expression, we observed a significant increase in the levels of *Ifng* and, to a lesser extent, *Tbx21* in TGF-β1-deficient Treg cells compared to those of TGF-β1-sufficient Treg cells. In addition, we observed a significant increase in the level of *Il12rb2* expression in the TGF-β1-deficient Treg cells while that of *Il12rb1, Ifngr1, Ifngr2, Il27ra*, and *Il6st* remained comparable (Figure 4B). These findings prompted us to hypothesize that autocrine TGF-β1 suppresses the expression of IL-12Rβ in Treg cells. To test this hypothesis, FACS-purified Treg cells were stimulated with anti-CD3ε and IL-12 in the presence of titrated doses of exogenous TGF-β1 for three days in vitro. Upon IL-12 stimulation, TGF-β1-deficient Treg cells expressed a higher level of *Il12rb2* than TGF-β1-sufficient Treg cells. The addition of exogenous TGF-β1 significantly suppressed the expressions of both *Il12rb1* and *Il12rb2* in Treg cells in a dose-dependent manner regardless of the TGF-β1-producing capacity of Treg cells. The difference in the expression of *Il12rb2* between TGF-β1-deficient and TGF-β1-sufficient Treg cells was no longer observed in the exogenous TGF-β1-treated cells (Figure 4C).

**Figure 5:**
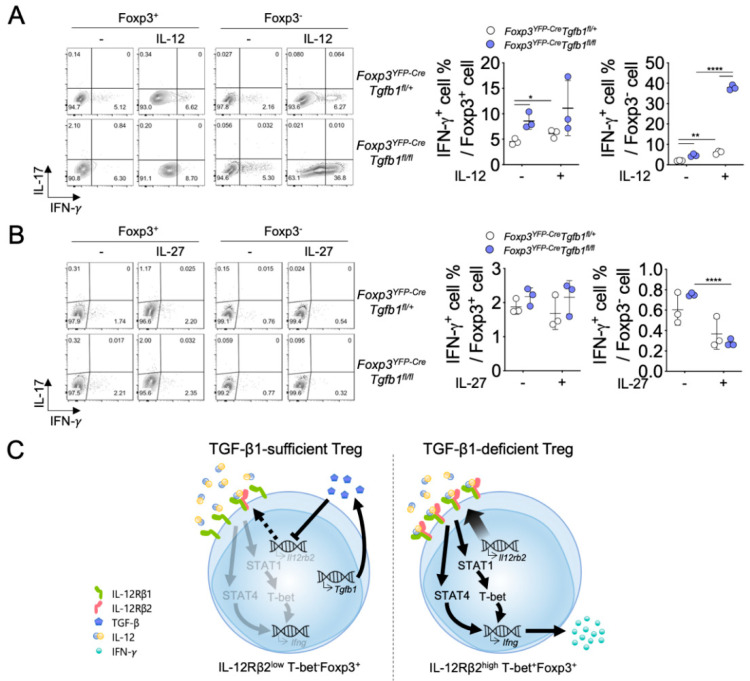
Ablation of TGF-β1 induces excessive IFN-γ production of Treg cells after IL-12 stimulation. (**A**) Sorted Treg cells from *Foxp3^YFP-Cre^Tgfb1^fl/+^* and *Foxp3^YFP-Cre^Tgfb1^fl/fl^* mice were stimulated with anti-CD3ε with (+ group) or without IL-12 (− group). Representative contour plots and quantification of IFN-γ- and IL-17A-expressing cells. (**B**) Sorted Treg cells from *Foxp3^YFP-Cre^Tgfb1^fl/+^* and *Foxp3^YFP-Cre^Tgfb1^fl/fl^* mice were stimulated with anti-CD3ε with (+ group) or without IL-27 (− group). Representative contour plots and quantification of IFN-γ- and IL-17A-expressing cells. Data are representative of four (**A**,**B**) independent experiments, and values are expressed as the mean ± SD (**A**,**B**). * *p* ≤ 0.05, ** *p* ≤ 0.01, and **** *p* ≤ 0.0001 compared to TGF-β1-sufficient Treg cells based on Student’s *t*-test. (**C**) Schematic model of the proposed mechanism.

To further define the pathways of TGF-β1-mediated regulation of IFN-γ production in Treg cells, we stimulated TGF-β1-sufficient and TGF-β1-deficient Treg cells with anti-CD3ε alone or with anti-CD3ε plus IL-12 or anti-CD3ε plus IL-27 for three days. While the TCR stimulation by anti-CD3ε marginally induced IFN-γ^+^ cells from TGF-β1-sufficient Treg cells, the addition of exogenous IL-12 significantly enhanced the induction of IFN-γ^+^ cells (Figure 5A). In contrast, exogenous IL-27 had a minimal effect on the induction of IFN-γ^+^ cells from TGF-β1-sufficient Treg cells (Figure 5B). TGF-β1-deficient Treg cells stimulated with anti-CD3ε alone contained a marginally increased frequency of IFN-γ^+^ cells compared to TGF-β1-sufficient Treg cells. However, TGF-β1-deficiency in Treg cells potentiated the induction of IFN-γ^+^ cells from Treg cells in the presence of exogenous IL-12, resulting in higher frequencies of IFN-γ^+^ cells in TGF-β1-deficient Treg cells compared to TGF-β1-sufficient Treg cells among Foxp3^+^ populations as well as among Foxp3^−^ populations. The augmentation of IFN-γ production in TGF-β1-deficient Treg cells was not observed with the addition of IL-27, suggesting that the IL-12 signaling pathway is specifically involved in the TGF-β1-regulation of IFN-γ expression in Treg cells (Figure 5C). Taken together, these results demonstrate an essential role for autocrine TGF-β1 in the maintenance of Treg cell stability via repression of IL-12-dependent IFN-γ expression in Treg cells.

## 4. Discussion

Immunosuppressive function of Treg cells is crucial for the maintenance of immune homeostasis and for the prevention of autoimmunity. Although Treg cells had been thought to be stable, recent studies have shown that they can be converted into effector-like CD4^+^ T cells under certain pathological conditions in vivo [26,29]. Given the higher self-reactivity of Treg cells than conventional CD4^+^ T cells [48], reprogramming of Treg cells into Th1-, Th2-, Th17- or Tfh-like effector cells could be detrimental to the host. The molecular mechanism underlying the regulation of Treg cells’ trans-differentiation into cytokine-producing effector-like cells is incompletely understood. Our findings strongly suggest that autocrine TGF-β1 inhibits trans-differentiation of Treg cells into Th1 cells by down-regulating the expression of IL-12Rβ.

While the role of TGF-β signaling in the thymic development of Treg cells is still debatable [20,21,22], TGF-β signaling in Treg cells has been shown to have a role in the regulation of thymic and peripheral expansion of Treg cells [20,23,49]. In addition, autocrine TGF-β1 is known to be responsible for the control of Treg cell expansion in the thymus as well as in the periphery [20,23]. In line with these studies, we found that the frequency of Treg cells in the thymus was increased in T cell-specific TGF-β1-deficient mice. However, we found that the frequencies of Treg cells in the thymus and the secondary lymphoid organs were comparable between *Foxp3^YFP-Cre^Tgfb1^fl/fl^* mice and littermate control mice, with an exception in the MLN where a marginal but significant decrease in Treg cells was observed in the former mice. Hence, we propose that autocrine TGF-β1 plays little role in the expansion of Treg cells in the primary and secondary lymphoid organs in vivo. By using Treg cells isolated from *Cd4creTgfb1^fl/fl^* mice, it has also been demonstrated that TGF-β1 producing capacity is required for Treg cells to inhibit the induction of Th1 cells from naïve CD4^+^ T cells in an animal model of T cell-mediated colitis [23]. By contrast, we observed that TGF-β1-deficient Treg cells isolated from *Foxp3^YFP-Cre^Tgfb1^fl/fl^* mice did not affect the Th1 cell induction from naïve CD4^+^ T cells but rather resulted in a significantly elevated conversion of Treg cells into IFN-γ^+^Foxp3^−^ cells from Treg cells in vitro and in vivo. Since severe spontaneous inflammation in *Cd4CreTgfb1^fl/fl^* mice might have affected the suppressive activity of TGF-β1-deficient Treg cells in the previous study, we propose that Treg cell-derived TGF-β1 may be more important for the suppression of Treg cell conversion into IFN-γ^+^Foxp3^−^ exTreg cells than for the suppression of Th1 cell induction from naïve CD4^+^ T cells in vivo. A recent study showed that *Tgfbr1*-deficient Treg cells exhibit elevated Th1 phenotype (T-bet expression) and reduced Th17 phenotypes (RORγt and IL-17 expression), supporting the notion that autocrine TGF-β1 is required for the suppression of Th1 phenotype in Treg cells [25].

A fraction of Treg cells can lose Foxp3 expression with a concomitant acquisition of Th1-like effector phenotypes under type 1 inflammatory conditions. It has been suggested that these ‘exTreg Th1 cells’ contribute to the pathogenesis of certain autoimmune diseases including T1D and experimental autoimmune encephalomyelitis [26,29]. In contrast, Treg cells can acquire Th1-like effector phenotypes while maintaining their Foxp3 expression, resulting in the development of IFN-γ^+^Foxp3^+^ Th1-like Treg cells. These Th1-like Treg cells are generated through the activation of the PI(3)K-Akt-FoxO pathway, which seems to be mediated by IFN-γ, IL-12 or IL-27 stimulation [50]. Th1-like Treg cells are also observed in various disease settings in mice and humans; however, their role in the pathogenesis of the diseases remains obscure [32,50,51,52,53,54]. In addition, whether the Th1-like Treg cells are an intermediate cell population during the development of exTreg Th1 cells from Treg cells under type 1 inflammatory conditions or whether the two are developmentally independent is still unclear.

The molecular mechanisms by which the stability of Treg cells is regulated under homeostatic conditions have been poorly understood. In the present study, we convincingly demonstrate an essential role for autocrine TGF-β1 in maintaining the stability of Treg cells under lymphopenic or inflammatory conditions. In an in vitro Treg conversion assay, autocrine TGF-β1 was required to suppress Treg cell conversion into both exTreg Th1 cells and Th1-like Treg cells. In contrast, while we observed an increased frequency in exTreg cells among TGF-β1-deficient donor Treg cells in comparison to TGF-β1-sufficient donor Treg cells under lympopenic conditions in vivo, the induction of Th1-like Treg cells was comparable between the two donor Treg cells. Considering the contribution of a variety of local environmental factors that Treg cells encounter under lymphopenic conditions in vivo, further studies are required to dissect the environmental factors that induce the instability of Treg cells and their association with Treg cell-derived TGF-β1. Also, given the importance of the methylation status of the Treg cell-specific demethylated region (TSDR) within the *Foxp3* locus for the maintenance of Treg cell stability [55], further studies are warranted to determine whether autocrine TGF-β1 controls Treg cell stability via inducing the demethylation of TSDR.

Expressions of Nrp1, ICOS, and PD-1 can differentially contribute to the function and stability of Treg cells. Recent studies demonstrate that Nrp1 expression is linked to the stability of Treg cells [56,57]. Consistent with these findings, our data showed that the frequency of Nrp1^+^ Treg cells was significantly decreased in the pLN of *Foxp3^YFP-Cre^Tgfb1^fl/fl^* mice. It remains unclear whether autocrine TGF-β1 controls Nrp1 expression in Treg cells and whether the Nrp1 down-regulation contributes to the instability of Treg cells in *Foxp3^YFP-Cre^Tgfb1^fl/fl^* mice. The expression of PD-1 and ICOS has been shown to be positively correlated with the suppressive activity of Treg cells [58,59]. However, although TGF-β1-deficient Treg cells harbored higher PD-1 and ICOS than TGF-β1-sufficient Treg cells, the former appeared to be less suppressive and more prone to produce IFN-γ than the latter. Hence, the expression of ICOS and PD-1 does not necessarily correlate with the suppressive activity of Treg cells, particularly in the absence of autocrine TGF-β1. The molecular mechanism by which autocrine TGF-β1 regulates the surface expression of ICOS and PD-1 remains to be determined.

T-bet directly interacts with the *Il12rb2* loci and positively regulates the expression of *Il12rb2* [60]. It has also been shown that TGF-β-mediated translocation of Smad2/3 into the nucleus downregulates T-bet expression in CD4^+^ T cells [20]. Given that T-bet^+^ Treg cells do not typically express IFN-γ due to the decreased expression level of IL-12Rβ2 compared to T-bet^+^ Th1 cells [61], Treg cell-intrinsic factors might have contributed to the down-regulation of IL-12Rβ2 and IFN-γ in Treg cells. In this regard, we found that deficiency of cell-intrinsic TGF-β1 promoted the expression of *Il12rb2* in Treg cells, while the supplementation with exogenous TGF-β1 repressed the up-regulation of *Il12rb2* expression in TGF-β1-deficient Treg cells. Collectively, these results suggest that autocrine TGF-β1 inhibits IFN-γ production in Treg cells by repressing the T-bet-dependent induction of IL-12β2 expression. In line with this notion, it has been shown that TGF-β inhibits the expression of IL-12R in murine splenocytes [62].

In summary, we found that autocrine TGF-β1 controls the reprogramming of Treg cells into IFN-γ^+^ cells via down-regulation of IL-12R signaling. Our findings provide mechanistic insights into understanding the regulation of Treg cell stability. Such insights could be useful in designing cellular therapeutics with Treg cells for the treatment of immune disorders in which improvement of Treg cell stability is crucial.

## Supplementary Materials

The following are available online at https://www.mdpi.com/2218-273X/10/6/819/s1, Figure S1: Expression of TGF-β1 in Foxp3^−^CD4^+^ and Treg cells, Figure S2: Treg cell phenotypes in control or T-cell specific TGF-β1-deficient mice, Figure S3: Expression of functional markers in Treg cells, Figure S4: IL-17 expression in TGF-β1-sufficient or deficient T cells.
