# Supplementary material for: Autocrine TGF-β1 Maintains the Stability of Foxp3+ Regulatory T Cells via IL-12Rβ2 Downregulation
Source: Biomolecules. 2020 May 27;10(6):819. doi: 10.3390/biom10060819 (PMC7356964; doi:10.3390/biom10060819)
Supplement: Supplementary file 1 [file biomolecules-10-00819-s001.pdf]

biomolecules

MDPI

Article-Supplementary Materials

Autocrine TGF-β1 maintains the stability of Foxp3$^+$ regulatory T cells via IL-12Rβ2 downregulation

Garam Choi[1,2], Hyeongjin Na[1,2], Da-Sol Kuen[1,2], Byung-Seok Kim[1,3,*] and Yeonseok Chung[1,2,*]

1 Laboratory of Immune Regulation, Research Institute of Pharmaceutical Sciences, College of Pharmacy, Seoul National University, Seoul 08826, Republic of Korea; grhappy@snu.ac.kr (G.C.); hyeongjinna@snu.ac.kr (H.N.); dkuen592@snu.ac.kr (D.-S.K.)

2 BK21 Plus Program, College of Pharmacy, Seoul National University, Seoul 08826, Republic of Korea

3 Division of Life Sciences, College of Life Science and Bioengineering, Incheon National University, Incheon 22012, Republic of Korea

* Correspondence: yeonseok@snu.ac.kr (Y.C.); Tel: +82-2-880-7874 (Y.C.); Fax: +82-2-872-1795 (Y.C.); byungseokkim@inu.ac.kr (B.-S.K.); Tel: +82-32-835-8893 (B.-S.K.); Fax: +82-32-835-0754 (B.-S.K.)

Received: date; Accepted: date; Published: date

Supplemental Figures

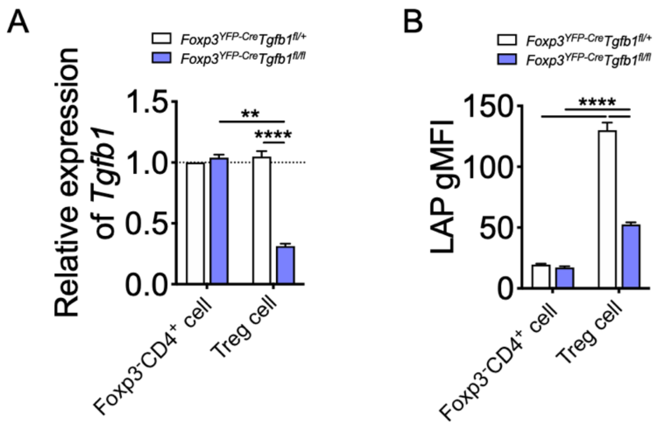

Supplemental figure 1. Expression of TGF-β1 in Foxp3$^-$CD4$^+$ and Treg cells. (A) Sorted Foxp3$^-$CD4$^+$ or Foxp3$^+$CD4$^+$ (Treg) cells from *Foxp3$^{YFP\text{-}Cre}$Tgfb1$^{fl/fl/+}$ or Foxp3$^{YFP\text{-}Cre}$Tgfb1$^{fl/fl}$* were analyzed by qRT-PCR. (B) Geometric mean fluorescence intensity (gMFI) of LAP among Foxp3$^-$ CD4$^+$cells or Treg cells from mesenteric lymph nodes (MLN) (n=4). Quantification plots show mean +SEM (A and B). **p≤0.01 and ****p≤0.0001 by two-tailed Student's t test.

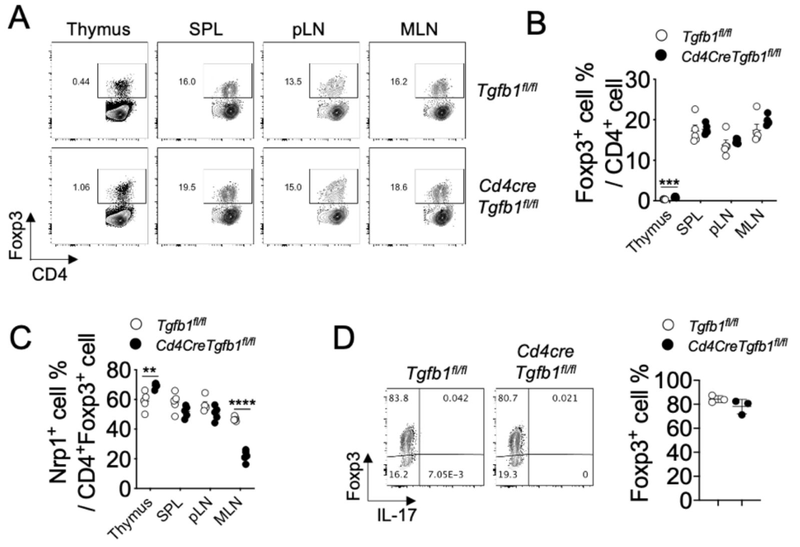

Supplemental figure 2. Treg cell phenotypes in control or T-cell specific TGF-β1-deficient mice. (A) Flow cytometric analysis of Foxp3 expression in CD4$^+$ T cells from thymus, spleen (SPL), peripheral lymph nodes (pLN) and MLN of *Tgfb1$^{fl/fl}$* and *Cd4CreTgfb1$^{fl/fl}$* mice at 8-15-week-old (n=5). (B) Graph depicting the frequency of Foxp3$^+$ cells among gated CD4$^+$ cells from thymus, SPL, pLN and MLN (n=5). (C) Expression of Nrp1 in CD4$^+$Foxp3$^+$ cells from thymus, SPL, pLN and MLN (n=4). (D) Naïve T cells from *Cd4creTgfb1$^{fl/fl}$* and *Tgfb1$^{fl/fl}$* mice were differentiated to Treg cells in vitro for 3 days and analyzed for the expression of Foxp3 by flow cytometry. Data are representative of four (A-C) or two (D) independent experiments. Quantification plots show mean ±SD (B-D). **p≤0.01, ***p≤0.001 and ****p≤0.0001 by two-tailed Student's t test.

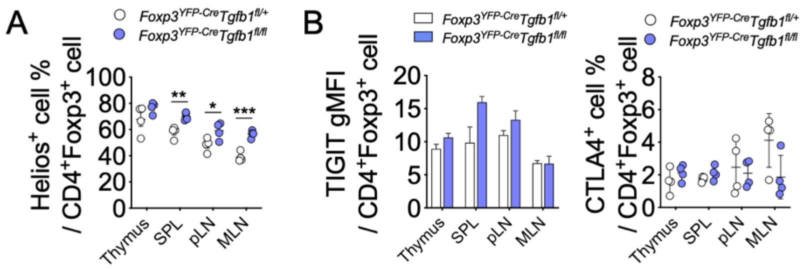

Supplemental figure 3. Expression of functional markers in Treg cells. (A) Frequencies of Helios+ cells in CD4+Foxp3+ cells from thymus, SPL, pLN and MLN (n=4). (B) Expression of TIGIT and CTLA4 in CD4+Foxp3+ cells from thymus, SPL, pLN and MLN (n=4). Quantification plots show mean ±SD (A, B; right graph) or +SEM (B; left graph). *$p \leq 0.05$, **$p \leq 0.01$ and ***$p \leq 0.001$ by two-tailed Student's t test.

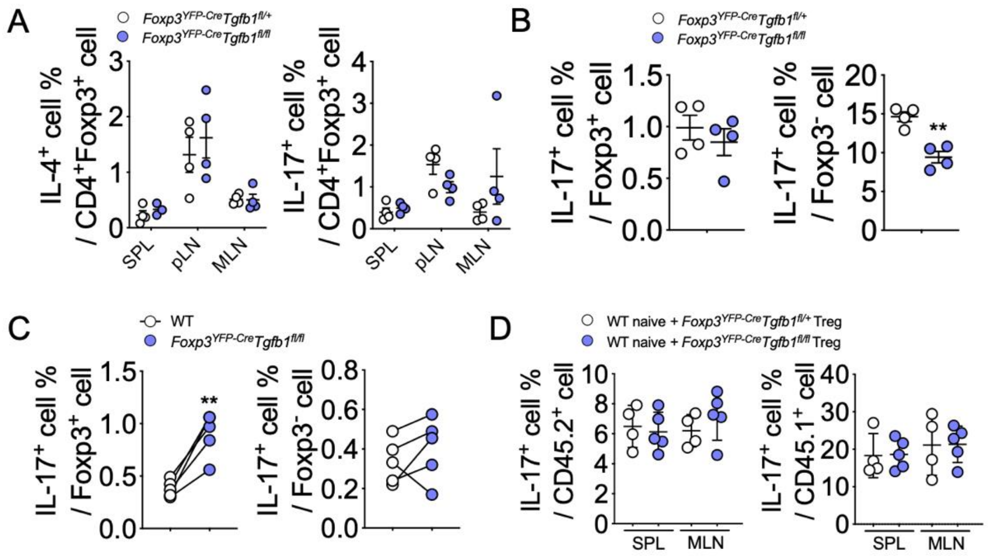

Supplemental figure 4. IL-17 expression in TGF-β1-sufficient or deficient T cells. (A) Flow cytometric analysis of IL-4 or IL-17 expression in CD4⁺Foxp3⁺ T cells from SPL, pLN and MLN of *Foxp3$^{YFP\text{-}Cre}$Tgfb1$^{fl/+}$* and *Foxp3$^{YFP\text{-}Cre}$Tgfb1$^{fl/fl}$* mice at 8-week-old (n=4) (B) Purified Treg cells co-cultured with bone marrow-derived dendritic cells (BMDC) in the presence of LPS. Quantification of IL-17-expressing cells among CD4$^+$Foxp3$^+$ or CD4$^+$Foxp3$^-$ cells. (C) Mixed-Treg cell co-culture experiment. Quantification of IL-17-expressing cells among CD4$^+$Foxp3$^+$ or CD4$^+$Foxp3$^-$ cells in CD45.1$^+$ (Wild-type (WT); TGF-β1-sufficient) or CD45.2$^+$ (TGF-β1-deficient) cells. (D) WT naïve T cells (CD45.1$^+$) were transferred to *Rag1$^{-/-}$* mice with sorted YFP$^+$Treg cells from *Foxp3$^{YFP\text{-}Cre}$Tgfb1$^{fl/+}$* or *Foxp3$^{YFP\text{-}Cre}$Tgfb1$^{fl/fl}$* mice. Quantification of IL-17$^+$ cells among CD45.1$^+$ cells in SPL and MLN (n=4-5). Data are representative of two (A, C, D) or five (B) independent experiments. Quantification plots show mean ±SD (A, B, D). **$p \leq 0.01$ by two-tailed Student's t test.
